# Public acceptance and expectations for Lecanemab: Insights from a Smallest Worthwhile Difference study

**DOI:** 10.1111/pcn.70082

**Published:** 2026-05-20

**Authors:** Aran Tajika, Kenji Omae, Ethan Sahker, Yan Luo, Yoshiteru Takekita, Toshi A. Furukawa

**Affiliations:** ^1^ Department of Health Promotion and Behavioral Sciences Kyoto University Graduate School of Medicine/School of Public Health Kyoto Japan; ^2^ Department of Innovative Research and Education for Clinicians and Trainees (DiRECT) Fukushima Medical University Hospital Fukushima Japan; ^3^ Population Health and Policy Research Unit, Center for Medical Education and Internationalization, Graduate School of Medicine Kyoto University Kyoto Japan; ^4^ Department of Next‐Generation Organ Transplantation The University of Tokyo Graduate School of Medicine Tokyo Japan; ^5^ Center for Medical Education and Internationalization Kyoto University Graduate School of Medicine Kyoto Japan; ^6^ Department of Neuropsychiatry, Faculty of Medicine Kansai Medical University Osaka Japan; ^7^ Kyoto University Office of Institutional Advancement and Communications Kyoto Japan

**Keywords:** Alzheimer's disease, cost–benefit analysis, lecanemab, patient preference, the smallest worthwhile difference

## Abstract

**Aim:**

To determine the smallest worthwhile difference (SWD) for lecanemab, a disease‐modifying drug for Alzheimer's disease (AD), among the general Japanese population using a benefit‐harm trade‐off method.

**Methods:**

We conducted an online survey with 658 participants to evaluate their preferences for the treatment effect of lecanemab, given its associated risks and high cost. We calculated SWD as the minimum required increase in the possibility of maintaining cognitive function (relative to the 50% without lecanemab) that patients would accept in exchange for the burdens of treatment (adverse effects, costs, other inconveniences). We examined the median SWD for two scenarios: a family member (SWD‐families) and others (SWD‐others), and analyzed subgroup differences.

**Results:**

The median SWD revealed a 15% increase for SWD‐families and SWD‐others. This value exceeds the drug's actual efficacy of an 8% increase. Notably, 17% of respondents reported zero SWD, driven by an intense fear of AD and possibly high expectations from media coverage. Including these participants, nearly half of the respondents considered the current effect worthwhile. No noticeable difference was found between the SWD‐families and SWD‐others.

**Conclusion:**

Based on Japanese clinical scenarios, the public's median expectation for lecanemab exceeds its efficacy, though its current benefit remains acceptable to nearly half the population. These findings underscore that the SWD is shaped by psychological drivers, including fear and hope, rather than just reasoned evaluation. Clinicians must manage these expectations, accounting for highly heterogeneous treatment preferences. Future research should account for emotional factors that can overshadow a rational assessment of treatment value.

The number of people with dementia is steadily increasing with the aging global population. Currently, an estimated 55 million individuals worldwide live with dementia, and 60%–70% of these people are believed to have Alzheimer's disease (AD).^1^ Out of Japan's total population of approximately 120 million, it is estimated that by 2025, there will be 4.72 million people aged 65 and older with dementia and 5.64 million people with mild cognitive impairment (MCI), the prodromal stage of dementia.^2^ AD progresses from MCI to mild, moderate, and severe stages. The Alzheimer's Disease Assessment Scale‐Cognitive Subscale (ADAS‐cog)^3^ is frequently used to assess symptoms, with scores ranging from 0 to 70. For patients with MCI or mild AD, a three‐point increase in the ADAS‐Cog score is considered the minimally important change, representing a clinically perceptible decline in cognitive function.^4^

AD is characterized by the cerebral deposition of amyloid‐beta (Aβ) protein. For decades, therapeutic strategies have focused on symptom management, with acetylcholinesterase inhibitors (donepezil, galantamine, and rivastigmine) and N‐methyl‐D‐aspartate receptor antagonists (memantine) to enhance neurotransmission and brain activity. However, these drugs do not address underlying pathologies. This paradigm shifted in 2021 with the FDA's accelerated approval of aducanumab, classified as a disease‐modifying drug, the first anti‐Aβ monoclonal antibody designed to target and clear accumulated Aβ in the brain. Subsequently, in 2023, lecanemab, another anti‐Aβ antibody, received approval for use in MCI and mild AD in the United States and Japan.^5^ To date, several papers have been published on the effects of lecanemab.^6^, ^7^ The results of a large‐scale randomized controlled trial (RCT) revealed that the significant change in ADAS‐cog after 18 months was 4.14 points for lecanemab, compared with 5.58 for placebo. However, the need for intravenous administration, frequency of side effects, and the substantial cost of lecanemab must be considered. Pretreatment confirmation of cerebral amyloid pathology *via* amyloid positron emission tomography (PET) scans or cerebrospinal fluid analysis is mandatory. Treatment involves biweekly intravenous infusions, which can be burdensome for patients and caregivers. The most frequent adverse reactions, including asymptomatic cases, observed were infusion‐related reactions (26.4%) and amyloid‐related imaging abnormalities (ARIA), including cerebral microhemorrhages (ARIA‐H) (17.3%) and edema or effusions (ARIA‐E) (12.6%).^6^ This necessitates a rigorous safety protocol involving regular magnetic resonance imaging to detect ARIA. In addition to the cost of diagnostic tests, the high price of lecanemab is a considerable obstacle to its treatment. In Japan, the estimated 12‐month cost, including one amyloid PET scan, eight brain magnetic resonance imaging (MRI) scans, and 26 infusions, is approximately 3,330,000 Japanese Yen (JPY) (22,200 US dollars [USD]). Even with health insurance, the out‐of‐pocket expenses were substantial. Japan has a system called the high‐cost medical expense benefit, under which amounts exceeding a set monthly limit on out‐of‐pocket medical costs are reimbursed. By utilizing this system, annual medical treatment payments could be reduced to a maximum of 144,000 JPY (960 USD). One report suggested that lecanemab may only be cost‐effective at an annual price of approximately 740,000 JPY (4900 USD).^8^ However, it is unclear how much efficacy the general public expects from lecanemab, given its side effects and costs.

The smallest worthwhile difference (SWD) is a measure of the preference of patients or the general public for a particular treatment. SWD is a concept that differs from minimally important changes. The SWD asks what treatment effect size is considered worthwhile compared to a treatment alternative. Conversely, the minimally important change evaluates the effect by comparing the treatment group's results before and after the intervention. The benefit‐harm trade‐off (BHTO) method^9^ was used to determine a person's SWD. First, the researchers described the disorder and explained the negative aspects of the treatment, such as side effects and costs. After informing the participants of the percentage of people whose symptoms would worsen if they did not receive treatment, the researchers asked a series of questions about the level of effectiveness they would expect from the treatment to accept the treatment burdens. There have been studies on SWD for ailments such as chronic lower back pain,^10^ lung disease,^11^ fall prevention,^12^ and depression^13^, ^14^; however, none exist for lecanemab. In this study, we aimed to determine SWD for lecanemab by surveying the general population, assessing their preference regarding the effects of its treatment while considering associated treatment burden, using an online questionnaire.

## Methods

### Internet survey procedure

The participants were recruited through Macromill, a company specializing in online research surveys. Macromill has a large number of research panels, with approximately 36 million people in Japan. Of the registered panels, those aged ≥20 years were targeted, with no other exclusion criteria. Macromill's research panel was registered based on various background characteristics. For this study, we prioritized recruiting individuals who had registered the characteristics of “having AD oneself” or “having a family member with AD.” Subsequently, participants without these characteristics were recruited. In Macromill's surveys, the fastest 3% of responses were automatically removed, as an extremely short completion time is highly indicative of a lack of careful consideration.

After providing participants with an online explanation of the study, informed consent was obtained, and the subsequent survey was administered to those who agreed to participate. This study was approved by the Kyoto University Ethics Committee (R4789).

### Content of the web survey

The content presented to participants was divided into the following parts: (the details are provided in the Data S1).

#### Explanation of AD and lecanemab (Data S1: Supplement 1a)

After providing a general explanation of AD progression, we described the mechanism of action, side effects (including asymptomatic cases), and lecanemab costs. We explained how the high‐cost medical expense benefit would cover a portion of the medical expenses. Finally, we explained that, without treatment, 50 of 100 people would maintain their cognitive function after 1 year (for details, see below).

#### Clinical scenarios (Data S1: Supplement 1b)

We developed two clinical scenarios to estimate the SWD: one where a participant or their family member had AD, and another where the disease affected someone else. All participants responded to both scenarios. Since lecanemab is an extremely expensive drug, we hypothesized that the SWD would differ for both scenarios. Specifically, we expected that participants' SWD for their family (SWD‐families) would be lower than their SWD for others (SWD‐others). In other words, we assumed that the participants would be more willing to accept lecanemab for a smaller benefit if it were for a family member.

#### Determining the no‐treatment alternative baseline scenario

To estimate the SWD, we specified the proportion of patients with AD who could maintain cognitive function for 12 months without treatment. We used data from the Clarity AD study.^6^ We read the change in ADAS‐cog scores at 12 months from the graph in this paper (lecanemab: 1.85 ± 6.86, placebo:3.20 ± 6.66) and converted the continuous variable into binary (“maintained” or “worsened”) using the minimal important change of three points for AD.^15^ The proportion of people who maintained their cognitive function at 12 months was 48.8% (376/770) in the placebo group and 56.7% (427/753) in the lecanemab group, and the odds ratio (OR) is 1.4. To create a scenario to determine SWD, we simplified the number from the placebo group and standardized the denominator to 100, resulting in 50% (50/100) of patients with AD maintaining their cognitive function within 12 months without treatment.

#### Questionnaire of the SWD estimation (Data S1: Supplement 1c)

**Fig. 1:**
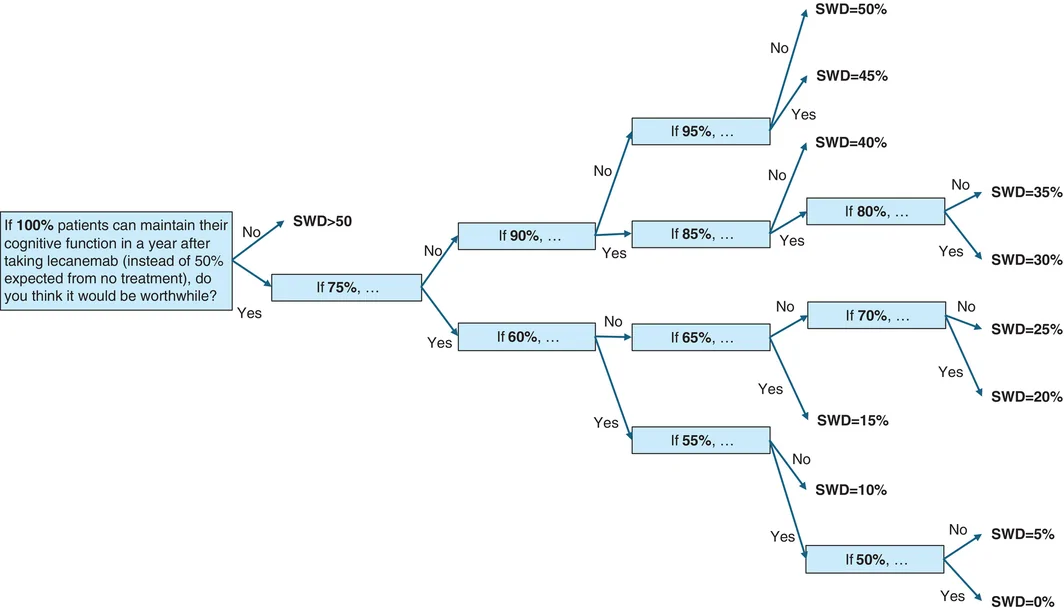
The algorithm of the SWD calculation.

After presenting the participants with the above no‐treatment situation (i.e., 50/100 maintained cognitive function), we used a sequence of questions to determine the SWD for lecanemab. The initial question asked participants whether they would choose lecanemab if it ensured that all of the 100 participants maintained their cognitive function. When the answer was “yes,” the percentage decreased for the next question; when the answer was “no,” the percentage increased. This process follows the algorithm shown in Figure 1. We asked questions based on this algorithm for each of the SWD‐families and SWD‐others. As hypothesized above, if individuals were willing to accept treatment for even a smaller effect in the family scenario, SWD‐families would be smaller than SWD‐others.

### Subgroup

We prespecified four subgroups based on whether they had family members with dementia and whether they had cohabited with patients with dementia. A: currently cohabitating with a family member with AD, B: previously cohabitated with a family member with AD; C: have a family member with AD but never cohabitated; and D: do not have a family member with AD. Experience with patients with dementia decreased from subgroups A to D. Individuals diagnosed with AD were included in subgroup A (currently cohabiting with a family member with AD) because in the treatment decision‐making process for AD, the preference of the patient and those of their close family are inseparable. Data collection continued until an equal number of participants were obtained for each of the four subgroups.

### Sample size calculation

In a previous study focused on depression,^13^ the median SWD was 25% (range: 0%–70%), with an interquartile range (IQR) of 10%–30% and a standard deviation (SD) of 18.5% (calculated as IQR/1.35). Given our study's SWD range of 0%–50%, we calculated a projected SD of 13.2% (18.5% × 50/70). We determined a necessary sample size of 170 participants per subgroup with a significance level of 0.05 and a 2% margin of error. With the four subgroups, our target population comprised 680 participants. We recruited participants and made adjustments as required to ensure that each subgroup had sufficient participants.

### Analysis

The SWD was set at 5% increments from 0% to 50% (Fig. 1). Participants who responded “no” to the first question were excluded from the analysis because it indicated they would refuse lecanemab treatment under any circumstances (i.e., their SWD was >50%). We calculated the median and distribution of the SWD‐families and SWD‐others. We also determined the percentage of patients satisfied with their current treatment effects. The effect size of lecanemab was calculated from the aforementioned Clarity AD study,^6^ where approximately 58% of patients on lecanemab maintained their cognitive function at 12 months compared with 50% without treatment, based on an OR of 1.4. We examined the distribution of SWD values for the four subgroups to compare differences between SWD‐families and SWD‐others. Statistical analyses were performed using R version 4.4.2.

### Protocol amendments and additional survey

Initially, we planned to perform the analysis using the method described above. However, we found that approximately 30% of the participants had an SWD of 0 in our first survey, indicating that they were willing to accept lecanemab treatment and its burdens, even if it had no effect. To investigate this, after 4 months, we readministered the same algorithmic questions to participants whose SWD (families or others) was zero. To avoid influencing participants' responses, we did not explain the rationale for readministering the same questions at that time. Participants whose SWD was still zero were asked to provide a free‐text explanation of their reasoning. We analyzed new responses from the participants who completed the follow‐up questions and excluded responses from those who did not.

## Results

In the initial survey conducted in March 2025, we received 704 responses. Of these, 236 (33.5%) reported a value of zero for SWD‐families, and 207 (29.4%) indicated zero for SWD‐others. In total, 250 individuals had at least one SWD score of zero. We conducted an additional survey among these individuals in July 2025 and received responses from 204 out of 250 participants (81.6%). We excluded the 46 nonrespondents, resulting in a final sample size of 658. The flow of participants from the initial survey to the additional survey is presented in Data S1: Supplement 2. Table 1 presents the characteristics of the included and excluded participants. Upon examining the participants, the sex distribution revealed that a higher percentage of males was in the cohabiting group, whereas a higher percentage of females was in the non‐cohabiting group. The participants had a median age in the mid‐50s, with a nearly equal distribution in all groups.

**Table 1:** Sociodemographic characteristics

		Included (*n* = 658)				
			Subgroups based on cohabitation experience with dementia patients			
	Excluded (*n* = 46)	Total	Currently cohabitating with a family member with AD (*n* = 166)	Previously cohabitated with a family member with AD (*n* = 162)	Have a family member with AD but never cohabitated (*n* = 166)	Do not have a family member with AD (*n* = 164)
Sex						
Male	26 (56.5)	345 (52.4)	120 (72.3)	89 (54.9)	47 (28.3)	89 (54.3)
Female	20 (43.5)	313 (47.6)	46 (27.7)	73 (45.1)	119 (71.7)	75 (45.7)
Age						
Mean ± SD	53.7 ± 11.6	55.5 ± 11.4	55.9 ± 11.2	57.0 ± 11.0	55.7 ± 11.0	53.5 ± 12.2
Median [IQR]	55.0 [47.8, 64.3]	57.0 [49.0, 63.0]	56.0 [50.0, 62.3]	58.0 [50.0, 65.0]	57.0 [51.0, 64.0]	54.0 [44.3, 61.8]
Minimum	28	21	23	21	24	26
Maximum	71	86	83	84	76	86
Marital status						
Unmarried	24 (52.2)	277 (42.1)	88 (53.0)	71 (43.8)	52 (31.3)	66 (40.2)
Married	22 (47.8)	381 (57.9)	78 (47.0)	91 (56.2)	114 (68.7)	98 (59.8)
Children						
No children	21 (45.7)	285 (43.3)	86 (51.8)	74 (45.7)	52 (31.3)	73 (44.5)
Has children	25 (54.3)	373 (56.7)	80 (48.2)	88 (54.3)	114 (68.7)	91 (55.5)
Employment status						
Full‐time	20 (43.5)	272 (41.3)	83 (50.0)	54 (33.3)	54 (32.5)	81 (49.4)
Part‐time	8 (17.4)	132 (20.1)	25 (15.1)	37 (22.8)	44 (26.5)	26 (15.9)
Seeking	4 (8.7)	18 (2.7)	9 (5.4)	3 (1.9)	1 (0.6)	5 (3.0)
Homemaker	3 (6.5)	98 (14.9)	12 (7.2)	27 (16.7)	40 (24.1)	19 (11.6)
Retired	4 (8.7)	74 (11.2)	21 (12.7)	24 (14.8)	11 (6.6)	18 (11.0)
Others	7 (15.2)	64 (9.7)	16 (9.6)	17 (10.5)	16 (9.6)	15 (9.1)
Education level						
Junior high school	1 (2.2)	10 (1.5)	4 (2.4)	1 (0.6)	1 (0.6)	4 (2.4)
High school	13 (28.3)	200 (30.4)	49 (29.5)	49 (30.2)	47 (28.3)	55 (33.5)
Junior college	8 (17.4)	147 (22.3)	30 (18.1)	37 (22.8)	47 (28.3)	33 (20.1)
Bachelor's	22 (47.8)	270 (41.0)	76 (45.8)	71 (43.8)	62 (37.3)	61 (37.2)
Master's	2 (4.3)	24 (3.6)	6 (3.6)	4 (2.5)	7 (4.2)	7 (4.3)
Doctorate	0 (0)	4 (0.6)	1 (0.6)	0 (0)	2 (1.2)	1 (0.6)
Others	0 (0)	3 (0.5)	0 (0)	0 (0)	0 (0)	3 (1.8)
Household income (JPY)						
<2,000,000	9 (19.6)	57 (8.7)	12 (7.2)	15 (9.3)	12 (7.2)	11 (6.7)
2,000,000–4,000,000	9 (19.6)	129 (19.6)	38 (22.9)	35 (21.6)	38 (22.9)	31 (18.9)
4,000,000–6,000,000	2 (4.3)	119 (18.1)	27 (16.3)	27 (16.7)	27 (16.3)	31 (18.9)
6,000,000–8,000,000	2 (4.3)	93 (14.1)	22 (13.3)	25 (15.4)	22 (13.3)	22 (13.4)
8,000,000–10,000,000	7 (15.2)	62 (9.4)	20 (12.0)	12 (7.4)	20 (12.0)	13 (7.9)
10,000,000–12,000,000	2 (4.3)	18 (2.7)	7 (4.2)	4 (2.5)	7 (4.2)	5 (3.0)
12,000,000–15,000,000	1 (2.2)	21 (3.2)	5 (3.0)	9 (5.6)	5 (3.0)	4 (2.4)
15,000,000–20,000,000	1 (2.2)	3 (0.5)	2 (1.2)	0 (0)	2 (1.2)	1 (0.6)
≥20,000,000	0 (0)	7 (1.1)	3 (1.8)	0 (0)	3 (1.8)	2 (1.2)
Don't know	5 (10.9)	88 (13.4)	19 (11.4)	18 (11.1)	19 (11.4)	27 (16.5)
No answer	8 (17.4)	61 (9.3)	11 (6.6)	17 (10.5)	11 (6.6)	17 (10.4)

AD, Alzheimer's disease; IQR, interquartile range; JPY, Japanese Yen; SD, standard deviation.

### Distributions of SWD‐families and SWD‐others

Of the 658 participants, 138 (21.0%) were unwilling to pursue treatment in either scenario, even if they had 100% effectiveness and were assumed to have SWDs >50%. When separating the scenarios, 125 individuals had SWD‐families >50, whereas 115 had a SWD‐others >50. After excluding these participants, we calculated the distributions of SWD‐families and SWD‐others using 533 and 543 participants, respectively.

**Fig. 2:**
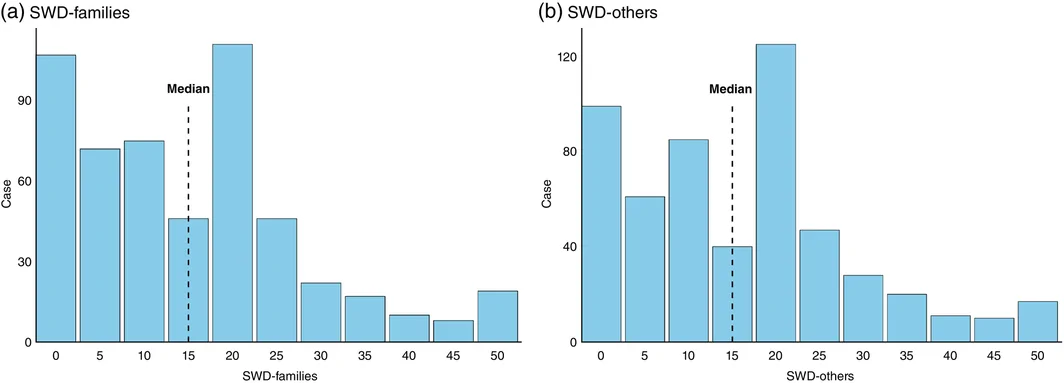
Distribution of SWD.

Figure 2a shows the distribution of SWD‐families. The distribution was bimodal, with the most frequent score being 20. Notably, a number of individuals reported a score of zero (*n* = 107), with a median of 15 [5, 20]. Given that lecanemab increases the proportion of people maintaining cognitive function by 8%, we can conclude that participants with an SWD‐families score of ≤10 are almost satisfied with the drug's current effect size. This group comprises 254 individuals, accounting for 47.7% of the total population.

Figure 2b shows the distribution of the SWD‐others. Of these individuals, 99 had a score of zero for SWD‐others, with a median score of 15 [5, 20]. The distribution was similar to that of the SWD‐families. The percentage of people satisfied with the current treatment effect size (SWD‐others ≤10) was 45.1% (245/658).

### Subgroups based on cohabitation experience with patients with AD

**Fig. 3:**
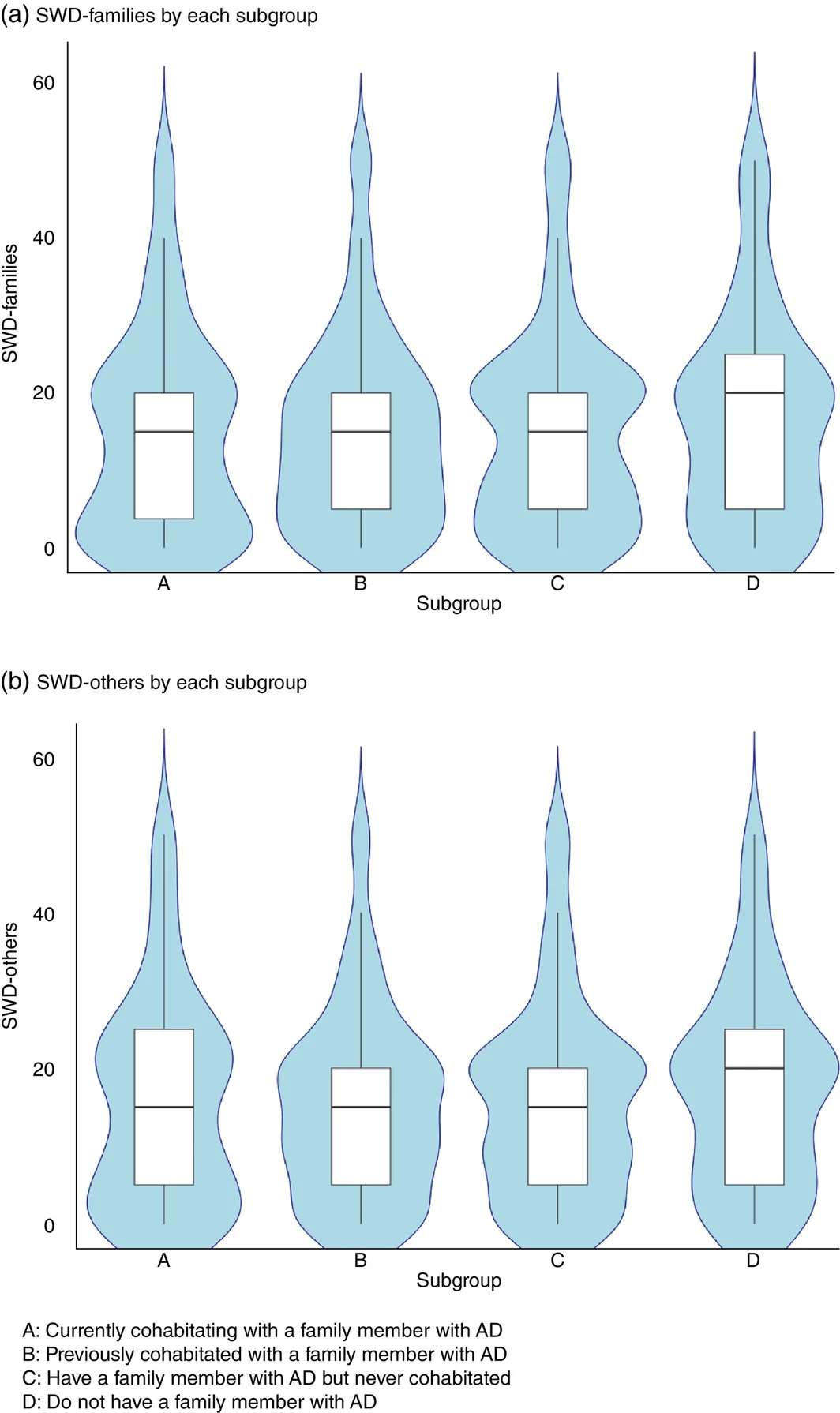
Subgroups based on cohabitation experience with AD patients.

Figure 3 shows the distribution of SWD, categorized into subgroups based on living with a family member with AD (Fig. 3a: SWD‐families; Fig. 3b: SWD‐others). Both distributions were very similar. Looking at the median values for each subgroup, only the “Do not have a family member with AD” group had an SWD score of 20 for SWD‐families and SWD‐others, whereas the other groups had a score of 15.

### Differences between SWD‐families and SWD‐others

We analyzed the treatment preferences of 138 individuals who scored >50 on either the SWD‐families or SWD‐others, indicating that they were unwilling to pursue lecanemab treatment in either scenario. The results revealed that 102 of these individuals did not want treatment in either scenario. Thirteen individuals were willing to have treatment in the “families” scenario but not in the “others” scenario. Conversely, 23 individuals were willing to have treatment in the “others” scenario but not in the “families” scenario. Overall, no noticeable differences were observed between the two scenarios.

**Fig. 4:**
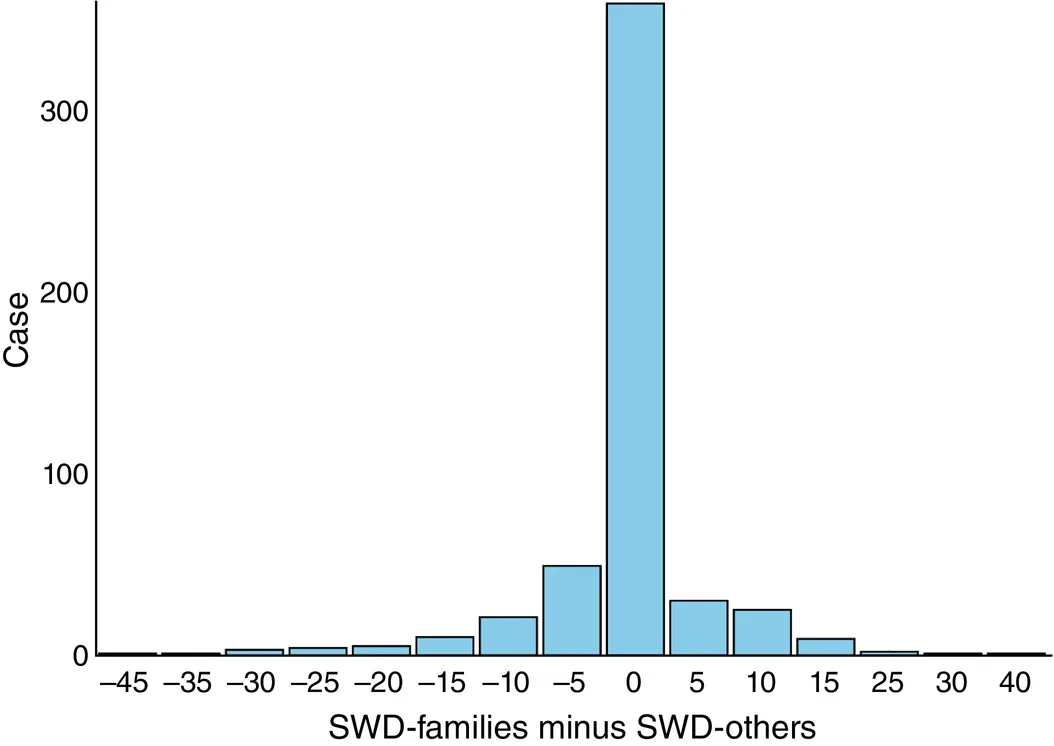
Differences between both SWDs.

Next, we analyzed 520 participants with scores of ≤50 for SWD‐families and SWD‐others. Figure 4 shows the distribution of the differences between these two scores (SWD‐families minus SWD‐others). The results indicated that 358 participants had identical scores. Ninety‐four participants had SWD‐families < SWD‐others, and 68 had SWD‐families > SWD‐others. The distribution was symmetrical around zero, contrary to the initial hypothesis that SWD‐family scores would be lower than SWD‐other scores.

### Reasons for reporting zero SWD

Of the 250 people in the additional survey, 111 (44%) had an SWD‐families or SWD‐others value of zero. Responses demonstrating clear misunderstandings of the numerical values or ambiguous answers such as ‘I don't know’ or ‘no reason’ were excluded. This resulted in 96 valid reasons for SWD‐families and 88 for SWD‐others. A summary of the reasons for hoping for lecanemab treatment even without an effect is listed in Table 2. Many of these reasons revolve around hope for a new drug, fear of AD, and the desire to avoid regret.

**Table 2:** The summaries of the reasons for hoping lecanemab despite a zero SWD

	SWD‐families (*n* = 96)	SWD‐others (*n* = 88)
Betting on possibility or hope	59 (61.5)	50 (56.8)
Fear of dementia and burdening family	23 (24.0)	17 (19.3)
Maintain status quo to avoid regret	17 (17.7)	16 (18.2)
A sense of security	8 (8.3)	8 (9.1)
Social perspective and other reasons	6 (6.3)	8 (9.1)

Duplicate responses included.

SWD, smallest worthwhile difference.

## Discussion

An internet survey with 658 participants revealed that 138 people (21%) would not take lecanemab even if it were 100% effective. Conversely, 111 people (17%) would take the treatment even if it had no effect. The main reason is fear of developing AD. Among those willing to consider lecanemab to prevent worsening of dementia, the median SWD was 15%. This means that they were willing to accept the burdens such as side effects, costs, and other inconveniences of receiving the lecanemab treatment if the chances of maintaining their cognitive function over 12 months were greater by 15% or more than that of 50% when not receiving lecanemab. Slightly less than half of the participants were satisfied with the current effect size of lecanemab treatment. There was no noticeable difference between SWD‐families and SWD‐others. The subgroup analysis revealed that the group with no experience of having a family member with AD had a median SWD of 20. Indicating that individuals without a patient in close proximity were less willing to pursue lecanemab treatment compared with those with a patient nearby, regardless of their cohabitation status.

We hypothesized that there would be a difference in the SWD between the two scenarios; however, no difference was found. Research has shown that caregivers of patients with dementia have a higher degree of emotional empathy than caregivers of stroke patients or non‐caregivers.^16^ Three‐quarters of the respondents had a close connection to patients with AD, regardless of cohabitation status. Therefore, they may have responded to other scenarios with the same mindset as they would have for their families. In a rapidly aging society, it is desirable to disseminate knowledge about dementia treatment strategies in a way that makes many people feel it is not “someone else's problem.”

Fear of the disease can influence decision‐making regarding AD treatment. AD is often depicted in literature and films as a terrifying, incurable disease.^17^ AD fear is centered on losing one's sense of self‐determination or autonomy.^18^ One study suggested that personal experiences, such as having a close relative with AD, are related to the perceived threat of the disease.^19^ Based on the results of a national survey in the United States about fear of various diseases, cancer ranked first at 40.3%, followed by dementia, including AD, at 17.5%.^20^ This study also reported that the fear of developing depression was only 1.4%, indicating that the fear of AD is exceptionally high compared with other neuropsychiatric disorders.^20^ SWD values can vary by disease and also by various other factors, making a simple comparison between diseases difficult. However, a study on the SWD of antidepressants revealed that only 1.9% (2/104) of respondents desired treatment despite it being ineffective, with modes of 10 and 30, while the median SWD was 20.^13^ This indicates that very few individuals are desperate for antidepressant treatment. This result supports the difference in fear towards AD and depression.

However, there may have been excessive expectations for lecanemab influenced by media coverage. Between June 2021 and September 19, 2025, the term “lecanemab” was mentioned in 87 articles in The Asahi Shimbun^21^ (Shimbun means newspaper in Japanese) and 142 articles in The Yomiuri Shimbun.^22^ It is rare for the name of a single drug to feature frequently in newspaper articles. Furthermore, donanemab received approval in September 2024, following lecanemab, and the associated media coverage may have influenced the survey responses. It is presumed that a considerable number of participants in this survey were familiar with the name of the drug. This may be a case in which heightened expectations for the drug led to a willingness to undergo treatment, even if it had no effect. The SWD score is an excellent tool for assessing patients' values and judgments regarding treatments based on excessive fear or expectations.

To determine the effect at the 12‐month mark, we used a 1.85‐point change in the ADAS‐cog for lecanemab from the Clarity AD study.^6^ Based on studies with a follow‐up period of at least 6 months, an ADAS‐cog change of 1.35 points for lecanemab was reported in a meta‐analysis.^23^ If this value is closer to the true effect, it would mean that the number of people who benefit from lecanemab treatment is much smaller than the increase from 50 to 58 per 100 individuals, as was initially assumed. Our study revealed that approximately half of the participants were satisfied with the current treatment effect; however, the proportion of satisfied participants would be smaller if that premise were to change. From a societal perspective, a simulation study in Japan revealed that by slowing the progression of the disease, lecanemab could reduce lifetime medical and long‐term care insurance costs by 1.99 million JPY (13,266 USD) per person, with a maximum value of 4.68 million JPY (31,200 USD).^24^ In this study, the estimation of the effect used data from the Clarity AD study.^6^ Subsequently, as we obtain more results from RCT, we will accurately measure the therapeutic effect and health economic value of lecanemab.

The limitations of this study are as follows: First, the pricing of lecanemab and the available systems for its use vary by country. In Japan, the protocol for lecanemab, which includes intravenous administration every 2 weeks and imaging tests at specified intervals, is strictly defined and covered by national health insurance. Additionally, depending on the case, patients may utilize Japan's unique high‐cost medical expense benefit system. Therefore, the scenarios we developed are inherently dependent on these specific Japanese contexts. Consequently, it is unknown whether the research findings from Japan can be applied to other countries. However, given that the high costs of drugs and various examination fees are common worldwide, it is unlikely that the results from Japan are completely inapplicable to other countries. Second, the methodology for determining the SWD involves asking for respondent preferences based on scenarios constructed using effect sizes from the best available evidence at that time. Given the complexities of the series of questions to estimate the SWD in the BHTO method, it was not feasible to present alternative scenarios for sensitivity analysis; consequently, the SWD is inherently dependent on the effect sizes reported in the existing literature, and it may not account for the high heterogeneity in treatment preferences. Third, this was a web‐based survey that utilized a survey panel rather than in‐person interviews. Therefore, the accuracy of the obtained results may be limited. However, this method is the only approach to recruit a large number of participants for this topic. Fourth, we did not anticipate that many participants would answer with an SWD of zero. To understand the reasons for this, we modified the protocol and conducted an additional survey. Consequently, we excluded those who did not respond to the additional survey. If we had anticipated this from the beginning and had included all the questions in the first survey, the results might have been slightly different. Nonetheless, the number of nonrespondents was small, and we were able to identify the reasons for this, which adds value to our study. Finally, although the values for SWD‐families and SWD‐others were very similar, presenting both scenarios consecutively may have caused the responses to be similar. In future investigations, the study population should be divided into two groups, with each group responding to only one scenario.

Conversely, the strength of the present study would include the following. First, we estimated the SWD of a new disease‐modifying drug for the first time. We followed a standard procedure (BHTO) to establish the SWD. As similar types of drugs are expected to be launched in the future, these results will serve as a useful reference regarding the preferences of the general population. Second, we surveyed a large group of community residents and collected sufficient samples for subgroup comparisons. Third, by adding an open‐ended question to individuals who reported an SWD score of zero, we were able to understand their reasons. Future SWD studies should incorporate open‐ended responses to examine the details of the extreme responses.

In conclusion, this study provides the first estimate of the SWD for lecanemab, developed within the specific context of Japan's clinical and insurance systems, and revealed a median SWD of 15% among the Japanese public. Importantly, our findings suggest that the SWD is not merely a reflection of clinical data but is significantly influenced by psychological factors, such as an intense fear of AD and high expectations potentially fueled by media coverage. This is evidenced by the substantial number of respondents who would accept the treatment even with zero efficacy, reflecting the highly heterogeneous nature of treatment preferences. These results highlight that clinicians must look beyond the numbers to manage patient expectations, and future research should further explore the emotional and psychological drivers that can overshadow a purely rational evaluation of treatment value.

## Author contributions

A.T. and T.A.F. conceived the study. A.T., T.A.F., ES, LY, and K.O. designed this study. Additional advice from a clinical perspective was provided by Y.T. A.T. performed the statistical analyses and had direct access to the full data. A.T. wrote the first draft. All authors contributed to the interpretation and critical revision of the manuscript and approved the final manuscript.

## Disclosure statement

A.T. received personal fees from Eisai and Shionogi outside the submitted work. Y.L. is employed at the Department of Next‐Generation Organ Transplantation, The University of Tokyo Hospital, which is an endowed department supported by the N28 General Incorporated Association, a nonprofit organization in Japan. Y.T. reports grant funding from the Japan Society for the Promotion of Science (23K07024); speaker honoraria from Meiji‐Seika Pharma, Sumitomo Pharma, Janssen Pharmaceutical, Otsuka, Eisai, Lundbeck, Daiichi Sankyo, Takeda Pharmaceutical, UCB Japan, Novartis, Teijin Pharma, Boehringer Ingelheim, EA Pharma, and Mitsubishi Tanabe Pharma; and consulting fees from Otsuka Holdings, IQVIA Japan, and Mitsubishi Tanabe Pharma outside of the submitted work. T.A.F. reports personal fees from Boehringer Ingelheim, Daiichi Sankyo, DT Axis, Micron, Shionogi, SONY, and UpToDate, and a grant from DT Axis and Shionogi, outside the submitted work; In addition, T.A.F. has a patent 7448125 and a pending patent 2024–521973 and has licensed intellectual properties for Kokoro‐app to DT Axis.

## Supporting information


**Data S1.** Supporting Information.

## Data Availability

The data that support the findings of this study are available from the corresponding author upon reasonable request.
