# Supplementary material for: Public acceptance and expectations for Lecanemab: Insights from a Smallest Worthwhile Difference study
Source: Psychiatry Clin Neurosci. 2026 May 20;80(9):740–7. doi: 10.1111/pcn.70082 (PMC13537328; doi:10.1111/pcn.70082)
Supplement: Supplementary file 1 [file PCN-80-740-s001.docx]

**Supplementary file**

**Supplement 1:**

**1a. Explanation of Alzheimer's disease and lecanemab (English translation)**

**1b. Clinical scenario (English translation)**

**1c. Questionnaire of the SWD estimation (English translation)**

**Supplement 2: Participant Flow from Initial Survey to Additional Survey**

**1a. Explanation of Alzheimer's disease and lecanemab (English translation)**

The number of people suffering from dementia is increasing rapidly, and it is estimated that in 2025, of the 36.5 million elderly people aged 65 and over, 4.72 million will have dementia and 5.64 million will have mild cognitive impairment (MCI), a pre-dementia stage of the disease.

A new drug called lecanemab (brand name leqembi) has just been launched for Alzheimer's disease, one of the most common types of dementia.

In this survey, we will begin by explaining Alzheimer's disease and lecanemab. After reading this, we will ask you how effective you would be willing to undergo (or have undergone) treatment with this drug. If you do not have someone close to you with Alzheimer's disease, please imagine "if I were to get Alzheimer's disease in the future" and answer the question.

You will be asked about two scenarios in turn: "If you or a family member receives treatment" and "If someone other than yourself or a family member receives treatment."

What are mild cognitive impairment (MCI) and Alzheimer's Disease?

There are several types of dementia, but Alzheimer's disease is the most common in the number of patients. In general, the disease progresses gradually from "mild cognitive impairment (MCI)" to "mild Alzheimer's disease" to "moderate Alzheimer's disease" to "severe Alzheimer's disease.

Mild cognitive impairment (MCI)

Mild	Alzheimer's disease

Moderate Alzheimer's disease

Severe	Alzheime's disease

- Mild cognitive impairment (MCI)

Although a little concerned about forgetfulness, the patient is leading an independent daily life. (At a level not diagnosed as dementia)

- Mild Alzheimer's disease

Losing track of dates. Failure to shop or cook.

- Moderate Alzheimer's disease

Losing track of where you are and getting lost. Become unable to choose appropriate clothing for themselves.

- Severe Alzheimer's disease

Can no longer recognize family members when they see them. Need assistance in all aspects of life.

As the disease progresses, the number of problems increases, and it is said that it is important to control the progression of the disease as much as possible.

It is known that one of the causes of Alzheimer's disease is the buildup of an unwanted protein called amyloid beta (beta) in the brain. To date, drugs have been marketed to slow the progression of Alzheimer's disease symptoms, but they have not been able to reduce amyloid beta.

<About the new treatment>

In 2023, a drug called "lecanemab" was launched that reduces amyloid beta. The characteristics of this drug are as follows.

- Lecanemab is intended for people with relatively mild symptoms, such as MCI and mild Alzheimer's disease.
- People with moderate or higher symptoms are not eligible.
- If symptoms worsen and become more than moderate, even with treatment, administration will be stopped there.
- To confirm that amyloid beta has accumulated in the brain, an amyloid PET (PET) scan (injecting a radiopharmaceutical that binds to amyloid and then imaging the brain) is performed before treatment begins.

- It is administered intravenously with a visit every two weeks.

- Occasional brain MRI scans are necessary to check for brain atrophy and side effects.

<Side effects>

There are two types of side effects: those associated with the infusion and those associated with the removal of amyloid beta from the brain. According to the results of clinical trials, the frequency with which side effects are seen is as follows

- Side effects of having an infusion (26%)

Headache, chills, fever, nausea, vomiting

- Side effects related to amyloid beta

Swelling of the brain (13%), cerebral hemorrhage (0.4%), microbleeding of the brain (14%), hemorrhaged blood adhering to the brain (5%)

<About the cost of one year's treatment>

For one year of treatment with lecanemab, the following laboratory and treatment fees will be required

- Amyloid PET scan: once before starting treatment
- Lecanemab infusion: 26 times
- Brain MRI scan: 8 times
- Bi-weekly office visit fee

The total is approximately 3.33 million yen (≈ 22,200 USD) per year.

The co-payment amount is about 1 million yen (≈ 6,666 USD) (about 83,000 yen (≈ 553 USD)/month) if 30% of the cost is paid.

- The cost will be approximately 670,000 yen (≈ 4,466 USD) (approximately 56,000 yen (≈ 373 USD)/month) if 20% of the cost is paid, and approximately 330,000 yen (≈ 2,200 USD) (approximately 28,000 yen (≈ 186 USD)/month) if 10% of the cost is paid.

However, under certain conditions, the "high-cost medical care benefits system" may apply, in which case the annual copayment will be a maximum of 144,000 yen (≈ 960 USD). In such cases, the difference will be covered by health insurance premiums and taxes.

- If treatment is continued beyond one year, depending on the duration, the cost of fortnightly lecanemab infusions, consultation fees, and periodic brain MRI scans will be charged.

If not treated with lecanemab

After one year, 50 out of 100 patients who do not receive treatment with lecanemab will experience a slight deterioration in cognitive function. Conversely, 50 out of 100 patients who do not receive treatment will remain the same without worsening symptoms.

- **Key points**
- **Lecanemab is administered intravenously once every two weeks.**
- **Treatment costs about 3.33 million yen (≈ 22,200 USD) per year, including the cost of testing.**
- **Using the high-cost medical care benefits system, the maximum annual cost is 144,000 yen** **(≈ 960 USD) (the difference is covered by health insurance premiums and taxes)**
- **50 out of 100 patients who do not receive treatment with lecanemab maintain the same condition one year later**

**1b. Clinical scenario (English translation)**

- Scenario 1

Suppose you or your family member is diagnosed with mild cognitive impairment (MCI) or mild Alzheimer's disease. If you do not receive treatment with lecanemab, the number of people who maintain cognitive function after one year is 50 out of 100. (The other 50 will have a slight decline in cognitive function.)

- Scenario 2

Suppose someone else, not related to you or your family, were to receive lecanemab through the high-cost medical care system. If he/she does not receive lecanemab, the number of people who maintain cognitive function after one year is 50 out of 100. (The other 50 will have a slight decline in cognitive function.)

**1c. Questionnaire of the SWD estimation (English translation)**

If lecanemab maintains cognitive function in 100 out of 100 people, do you think it would be worthwhile?

**No treatment**	**Lecanemab**

　　 　 50/100 maintain cognitive function 　 100 /100 maintain cognitive function

☐ Yes

☐ No

Please refer to Figure 1 for the algorithm of the subsequent questions.

**Supplement 2. Participant flow from initial survey to additional survey**
